# Next-generation des-r-carboxy prothrombin for immunohistochemical assessment of vascular invasion by hepatocellular carcinoma

**DOI:** 10.1186/s12893-020-00862-0

**Published:** 2020-09-14

**Authors:** Shintaro Yamazaki, Tadatoshi Takayama, Tomoharu Kurokawa, Naoaki Shimamoto, Yusuke Mitsuka, Nao Yoshida, Tokio Higaki, Masahiko Sugitani

**Affiliations:** 1 Departments of Digestive Surgery, Nihon University School of Medicine, 30-1 Ohyaguchikami-machi, Itabashi-ku, Tokyo, 173-8610 Japan; 2 Departments of Pathology, Nihon University School of Medicine, 30-1 Ohyaguchikami-machi, Itabashi-ku, Tokyo, 173-8610 Japan

**Keywords:** Des-r-carboxy prothrombin, Alpha-fetoprotein, Vascular invasion, Hepatocellular carcinoma

## Abstract

**Background:**

We have previously shown the value of next-generation des-r-carboxy prothrombin (NX-DCP) for predicting vascular invasion in hepatocellular carcinoma (HCC). Since conventional DCP is inaccurate under some conditions, this study aimed to assess whether NX-DCP immunohistochemical staining was related to vascular invasion in HCC.

**Methods:**

Fifty-six patients scheduled to undergo resection for single HCC were divided into two groups, with and without pathological portal vein invasion. Immunohistochemical features of HCC and sites of vascular invasion were assessed using alpha-fetoprotein (AFP), conventional DCP, and NX-DCP.

**Results:**

Pathological portal vein invasion was absent in 43 patients and present in 13 patients. Patient characteristics, pathological background of the liver parenchyma, and tumor-related factors did not differ significantly between the groups. There was no significant difference in the serum AFP level between the groups, whereas levels of conventional DCP (*p* < 0.0001) and NX-DCP (p < 0.0001) were significantly higher in the vascular invasion group. Immunohistochemical staining showed no significant difference in the staining rate of tumor (67.9% vs. 80.7%, *p* = 0.08), but NX-DCP stained significantly more at the sites of vascular invasion (15.4% vs. 46.2%, *p* = 0.01) than conventional DCP. No vascular invasion was stained by AFP.

**Conclusions:**

NX-DCP offers better sensitivity for detecting sites of vascular invasion than AFP and conventional DCP.

## Background

Des-r-carboxy prothrombin (DCP), known as protein-induced vitamin K absence or antagonist-II (PIVKA-II), is a biomarker for hepatocellular carcinoma (HCC) offering sensitivity of 40–56% and specificity of 81–98% [1–6]. The serum DCP level is considered more specific than alpha-fetoprotein (AFP) for malignant potential in HCC, but the diagnostic powers of the two markers do not overlap [6–8]. Conventional DCP has not become a commonly used marker because the value is not accurate in the presence of vitamin K deficiency, the use of anticoagulants, or poor nutritional status associated with alcoholic abuse or jaundice [9–12]. Therefore, the next-generation DCP (NX-DCP) was created to improve on the disadvantage of having to use two different antibodies (P-11 and P-16) [9–16]. The diagnostic accuracy of NX-DCP for HCC under various conditions has been shown in previous studies [9, 11, 15, 17]. NX-DCP is thus considered a useful biomarker for clinical use.

The local recurrence of HCC depends on tumor diameter, vascular invasion, and intrahepatic metastasis via the portal vein [2, 18, 19]. Of them, vascular invasion has the highest risk for recurrence [7, 18–20]. Therefore, anatomic resection for HCC had been performed to treat microvascular invasion [21]. However, there have been numerous studies of detecting microvascular invasion preoperatively, but there has not yet been a definitive study [22, 23].

Recently, owing to the development of gene analysis, HCC with microvascular invasion has been divided into less invasive and highly invasive phenotypes associated with two distinct gene-expression profiles [8]. Similarly, some studies have mentioned that the up-regulation of some genes and pathways such as aurora kinase B predicts a worse outcome [8, 24, 25]. These genes are closely related to vascular invasion. Thus, the clarification of the mechanisms underlying vascular invasion is important for determination of treatment strategies and early detection of recurrence.

We have already shown the better predictive value of NX-DCP for detecting vascular invasion compared to alpha-fetoprotein (AFP) [17]. That study showed that NX-DCP offered sensitivity of 71.4% and 1-specificity of 13.1% for predicting vascular invasion, with an area under the curve of 0.813. However, pathological information regarding NX-DCP remains limited, and no studies focusing on sites of vascular invasion as determined by immunochemistry have been reported. The aim of this study was to assess the value of NX-DCP for predicting vascular invasion in immunohistological studies.

## Methods

### Patients

Data of 61 patients who underwent liver resection for single pathologically confirmed HCC were collected. Five patients with HCC exceeding 50 mm in diameter were excluded, because large tumors have shown significant associations with vascular invasion [17]. Patients were divided into two groups, positive and negative for vascular invasion, according to the presence or absence of pathological portal vein invasion. Previous studies have shown that DCP levels were correlated with the degree of fibrosis, hepatitis, and warfarin pharmacotherapy [9–11, 16]. To avoid false positives, only patients with a single HCC less than 50 mm in diameter who were not on warfarin were examined.

### Tumor marker analysis

Blood samples (6 mL) were obtained from patients under general anesthesia before operation. The novel mouse anti-human DCP antibodies P11 (conventional DCP) and P16 (NX-DCP) were used. NX-DCP was analyzed from concentrated sera using the sandwich ECLIA method, with the assay system provided by EIDIA Co. (Tokyo, Japan) [10, 12]. Informed consent was obtained from all patients, and the study protocols were approved by the scientific committee in our hospital (RK-130308-12).

### Histopathological study

**Fig. 1:**
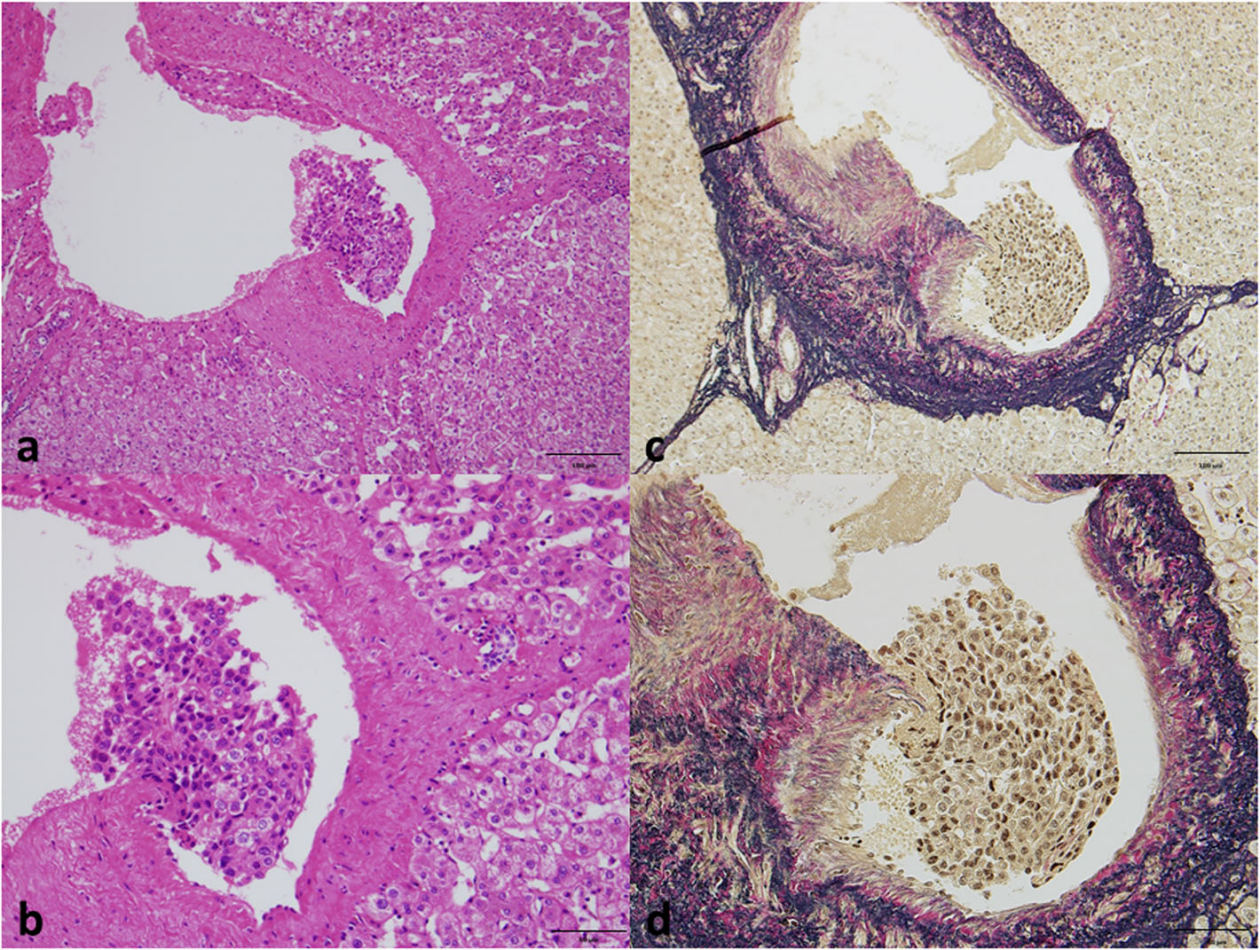
Elastica van Gieson staining for vascular invasion. When vascular invasion was suspected on hematoxylin and eosin staining (**a**, **b**), Elastica van Gieson staining was performed (**c**, **d**). (Original magnification: **a**, **c**; × 200, **b**, **d**; × 400)

All resected specimens were cut into 10-mm-thick slices. Slices showing visible vessels were sectioned longitudinally and fixed in 10% formalin. Two independent pathologists inspected these fixed specimens, and all possible metastases and areas of vascular invasion were trimmed for embedding in paraffin blocks. Next, 5-μm-thick sections for microscopy were stained with hematoxylin and eosin (HE). All areas of tumor were checked by two independent researchers (an expert pathologist, M.S., and a researcher for this study who was blinded to clinical data at inspection, T.K.) using HE staining to identify pathological portal vein invasion. Glisson’s sheath was found close to the tumor or was hard to discriminate from vascular invasion, so Elastica van Gieson staining was additionally performed (Fig. 1). Vascular invasion was defined by the presence of clusters of cancer cells in the vascular space linked by endothelial cells. When Glisson’s sheath was invaded and the structure was partially destroyed by cancer cells or the fibrous capsule surrounding the tumor was infiltrated by cancer cells, the serial microscopic sections were stained to confirm false positives. Regarding the evaluation of the background liver parenchyma, the New Inuyama classification was used to assess the degree of fibrosis in the liver (grade 0–4) and inflammation (grade 0–3) [26].

### Immunohistochemical staining

**Fig. 2:**
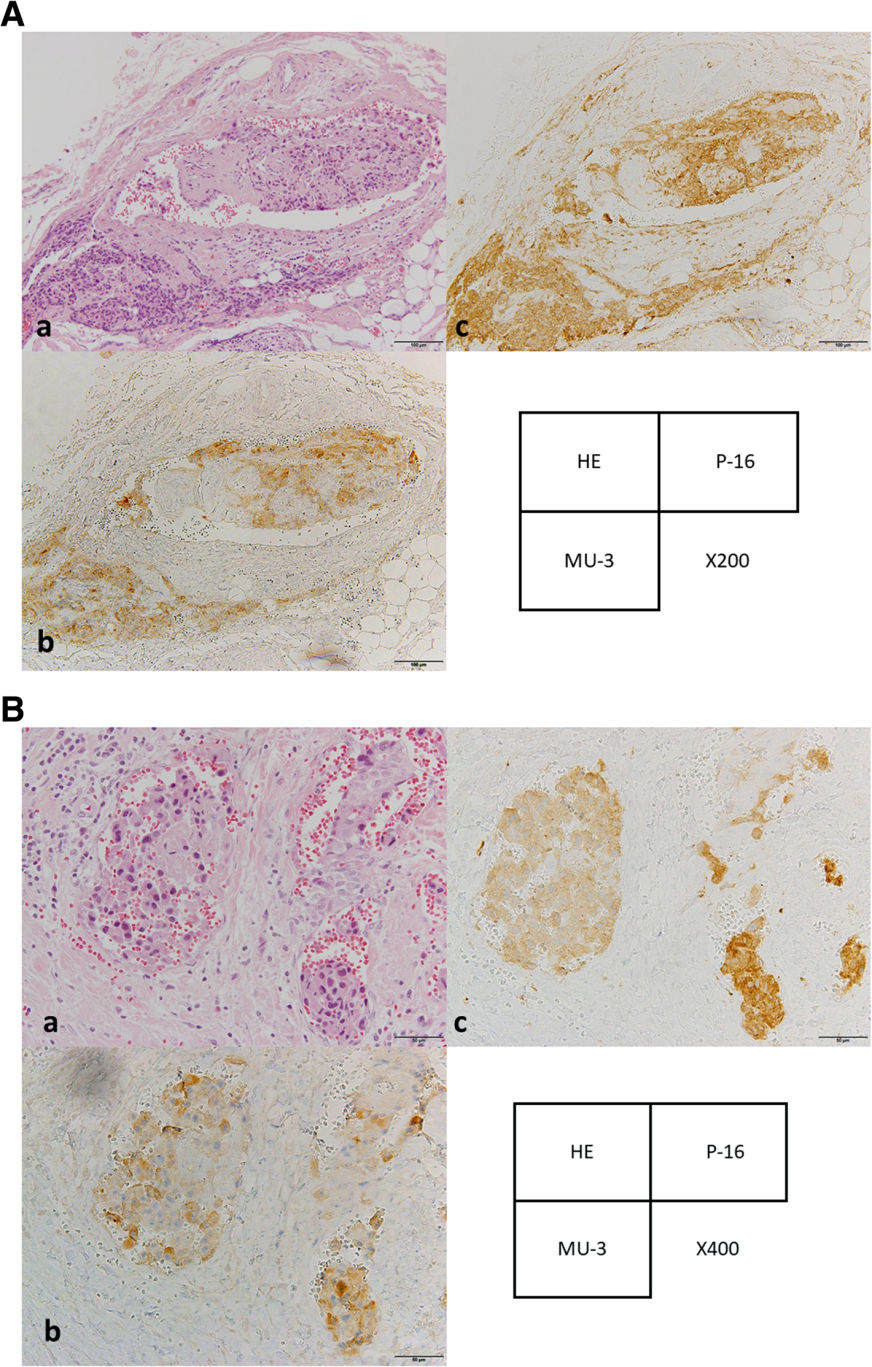
Immunohistochemical staining for vascular invasion sites. When vascular invasion was identified on HE staining, AFP staining, conventional DCP staining, and NX-DCP. The vascular invasion sites were positive for DCP and NX-DCP but negative for AFP staining. (Original magnification: 2A a-c; × 200, 2B a-c; × 400)

When vascular invasion was identified on HE staining, AFP staining, conventional DCP staining, and NX-DCP staining were added, 3 slides per patient (Fig. 2A, B). Immunohistochemical staining for tumor and at sites of vascular invasion was performed automatically using a Discovery XT system (Ventana Medical Systems, Tucson, AZ) using the Research IHC DAB MapXT procedure. Paraffin was melted at 70 °C for 30 min, followed by 10 min at room temperature. Slides were incubated for 1 h at 37 °C after a mild Ribo CC solution with a citrate-based buffer (pH 6.0) (Ventana Medical Systems). After rinsing, slides were incubated at 37 °C for 42 min with a 1:100 dilution of AFP, 1:100 dilution of conventional DCP (MU-3), or 1:250 dilution of next-generation DCP (P-16) (Eidia Co., Tokyo, Japan). Slides were incubated with biotinylated goat anti-mouse immunoglobulin (Ig) G and IgM secondary antibodies for 8 min, followed by incubation with proprietary Blocker D (Ventana Medical Systems) for 4 min. Slides were then counterstained for 4 min with hematoxylin and rinsed. After removal from the instrument, slides were manually dehydrated and coverslipped.

### Statistical analysis

Continuous variables were compared using Student’s *t*-test or the Mann-Whitney U test. Multiple comparisons were made using repeated-measures analysis of variance. Significance was defined as values of *p* < 0.05. All analyses were performed using JMP version 13.1 software (SAS, Chicago, IL).

## Results

### Comparison of patients’ characteristics by pathological vascular invasion

In the total of 56 participants, pathological portal vein invasion was present in 13 patients (positive vascular invasion group) and absent in 43 patients (negative vascular invasion group) during the study period. No significant differences in rate of hepatitis virus infection, basic liver function, platelet count, and coagulation activity were evident between the groups (Table 1). Regarding background liver parenchyma, no significant differences between groups were seen in either pathological grade of liver parenchyma (*p* = 0.07) or degree of hepatitis (*p* = 0.33).

**Table 1:** Patient characteristics according to vascular invasion

		Positive vascular invasion group	Negative vascular invasion group	*p* value
		(*n* = 13)	(*n* = 43)	
Gender	(male)	7 (53.9%)	26 (60.5%)	0.37
	(female)	6 (46.1%)	17 (39.5%)	
Age	(years)	70 (41–82)	73 (43–82)	0.81
HBV infection^a^		4 (30.8%)	15 (34.9%)	0.78
HCV infection^b^		8 (61.5%)	26 (60.5%)	0.94
Recurrence of hepatocellular carcinoma		5 (38.5%)	13 (30.2)	0.58
Aspartate aminotransferase	(IU/dL)	31 (17–84)	38 (14–139)	0.25
Alanine aminotransferase	(IU/dL)	26 (13–55)	36 (12–157)	0.26
Albumin	(g/dL)	4.1 (3.0–5.0)	4.0 (2.8–4.8)	0.36
Total bilirubin	(mg/dL)	0.72 (0.46–1.46)	0.72 (0.26–2.12)	0.94
Prothrombin activity	(%)	100 (82–100)	100 (68–100)	0.78
Platelets count	(mm^4^/dL)	15.3 (5.8–33.9)	11.5 (4.2–22.7)	0.08
Conventional DCP	(mAU/mL)	69 (8–3549)	22 (9–475)	0.04
Next-generation DCP	(mAU/mL)	74 (17–574)	30 (16–813)	0.01
Alpha-fetoprotein	(ng/mL)	9.7 (1.6–1208.7)	10.6 (1.6–453.2)	0.58
Pathological fibrosis grade^c^				0.07
f1		4	3	
f2		4	9	
f3		1	11	
f4		4	20	
Pathological hepatitis grade^c^				0.33
a1		4	13	
a2		4	15	
a3		4	15	
a4		1	0	
Tumor diameter	(mm)	28 (11–50)	22 (9–38)	0.06
Tumor differentiation				
Well-differentiated		0 (0%)	9 (20.9%)	0.02
Modelately differentiated		11 (84.6%)	30 (76.7%)	0.81
Poorly differentiated		2 (15.4%)	3 (7.0%)	0.38
Others		0	1 (2.3%)	0.41
Fibrous capsule infiltration of tumor		6 (46.2)	19 (44.2)	0.89

^a^ Hepatitis B virus; ^b^ hepatitis C virus; ^c^ according to the new Inuyama classification, *DCP* Des-r-carboxy prothrombin

As for tumor-related factors, diameter did not differ significantly, but it tended to be smaller in the negative vascular invasion group [median: 28 mm (ranged: 11–50 mm)] than in the positive vascular invasion group [median: 22 mm (ranged: 9–38 mm), *p* = 0.06]. Well-differentiated tumor was more frequent in the negative vascular invasion group (20.9%) than in the positive vascular invasion group (0%, *p* = 0.02), but no difference was identified in the frequency of poorly differentiated tumors (*p* = 0.38). No significant differences were apparent in the presence of fibrous capsule infiltration of the tumor (*p* = 0.89).

### Immunohistostaining of tumor and vascular invasion sites

Compared with the negative vascular invasion group, the positive vascular invasion group showed significantly higher levels of conventional DCP [median: 250 mAU/mL (range: 17–18,790 mAU/mL) vs. 31.0 mAU/mL (16–813 mAU/mL), respectively; *p* < 0.0001] and NX-DCP [median: 510 mAU/mL (range: 10–98,450 mAU/mL) vs. 34.0 mAU/mL (12–541 mAU/mL), respectively; p < 0.0001] (Table 1). In our previous study, the predictive cut-off value for vascular invasion of NX-DCP was 74 mAU/mL, with a sensitivity of 71.4%, and that of conventional DCP was 66 mAU/mL, with a sensitivity of 71.4% [17]. In contrast, no relationship was observed for the values of AFP, vascular endothelial growth factor, and vascular endothelial growth factor receptor with or without vascular invasion.

### Immunohistostaining grade of tumor and vascular invasion sites

According to immunohistochemical staining analyses of the original tumor, 38 of 56 patients (67.9%) showed positive staining for conventional DCP (MU-3), whereas 45 of 56 patients (80.4%) showed positive staining for NX-DCP (P-16) (Table 2). No significant differences in staining rates were evident regarding positive or negative for vascular invasion for both conventional DCP and NX-DCP. Among the 13 patients showing pathological portal vein invasion, conventional DCP identified a site of vascular invasion in 2 patients (15.4%), and NX-DCP identified vascular invasion in 6 patients (46.2%). In contrast, no patients showed vascular invasion sites with AFP staining. Regarding positive rates for staining, no significant differences were observed at the tumor between conventional DCP and NX-DCP (67.9% vs. 80.4%, respectively, *p* = 0.08). In contrast, significant differences were seen at sites of vascular invasion (15.4% vs. 46.2%, respectively, *p* = 0.01) (Table 3).

**Table 2:** Imunohistostaining of each biomarkers by site (*n* = 56)

	Positive vascular invasion group	Negative vascular invasion group	Total	*p* value^a^
Antibody	(*n* = 13)	(*n* = 43)	(*n* = 56)	
Tumor site				
Conventional DCP (MU-3)	9 (69.2%)	29 (67.4%)	38 (67.9%)	0.90
Next-generation DCP (P-16)	11 (84.6%)	34 (79.1%)	45 (80.4%)	0.66
Vascular invasion site				
Conventional DCP (MU-3)	2 (15.4%)	0 (0%)	2 (3.6%)	< 0.0001
Next-generation DCP (P-16)	6 (46.2%)	0 (0%)	6 (10.7%)	0.0008
Alpha-fetoprotein	0 (0%)	0 (0%)	0 (0%)	1.00

*DCP* Des-r-carboxy prothrombin. ^a^ Compared between portal vein (+) and pv(−)

**Table 3:** Rate of positive staining on immunohistochemistry by site

	Conventional DCP (MU-3)	Next-generation DCP (P-16)	*p* value
Tumor (*n* = 56)	38 (67.9%)	45 (80.4%)	0.08
Vascular invasion site (*n* = 13)	2 (15.4%)	6 (46.2%)	0.01

*DCP* Des-r-carboxy prothrombin

## Discussion

This study showed that the results of staining for NX-DCP (P-16) show a strong relationship to vascular invasion. Thus, together with our previous study, the serum NX-DCP values might offer accurate prediction of pathological vascular invasion [17].

Vascular invasion is a specific feature of highly invasive HCC and a predictor of recurrence and survival [7, 18, 20, 23]. Even though small HCC can be detected with recent advances in imaging modalities, only 10% of tumors with vascular invasion are detected by radiological diagnosis alone [22, 23]. It has been suggested that DCP offers better diagnostic power for vascular invasion compared to AFP [2, 3, 18–20]. Indeed, our previous study showed that 25.5% of patients with vascular invasion could be predicted by DCP [17]. Despite several reports on NX-DCP, none has yet confirmed a relationship between NX-DCP and vascular invasion.

AFP is a known tumor marker for HCC, and it has been reported to correlate with tumor diameter and differentiation of HCC [2–5]. The detection rate of HCC is 45% with AFP alone, increasing to more than 60% in combination with DCP [2, 3, 5, 17, 21]. However, no previous studies have shown that AFP is useful for detecting deleterious biological properties such as vascular invasion. In our previous study, no correlation was identified between the serum AFP value and vascular invasion [17]. Furthermore, no vascular invasion was positive on AFP staining in the present study. Use of AFP alone is thus insufficient for diagnosing and recognizing the malignant potential of HCC.

Previous studies have shown that NX-DCP levels were correlated with the degree of fibrosis and HCV infection [9–12]. NX-DCP expression was found in 82% of non-cancerous liver tissues, and overexpression was identified in patients with obstructive jaundice or on warfarin pharmacotherapy [16]. To avoid such false positives, only patients with a single HCC less than 50 mm in diameter who were not receiving warfarin were examined, and a staining grade based on background parenchyma was used in this immunohistochemical study. As a result, no strong correlations to degree of fibrosis or degree of hepatitis were found pathologically.

Recently, in analyses for gene overexpression, some pathways with microvascular invasion have been divided into low and highly invasive phenotypes associated with two distinct gene-expression profiles [16]. Vascular invasion reflects the biologically aggressive phenotype of HCC, because recurrence is frequent [18–20], and various molecules were up-regulated in the presence of vascular invasion [7, 16, 24, 27]. Tanaka et al. showed two distinct phenotypes in HCC by gene profiling [16]. They showed that the upregulation of the aurora kinase B-related pathway may be associated with the highly invasive phenotype of HCC by biomolecular network interaction analyses, and overexpression of aurora kinase B protein was confirmed by immunohistochemistry [16, 24]. In such studies, it was suggested that some component of DCP may be closely associated with the malignant phenotype of HCC. Therefore, further studies to search for biomarkers for the malignant population in HCC may lead to the development of novel, molecular-targeting therapies.

The limitation of this study is the small sample size, and the vascular invasion rates with conventional DCP (*n* = 2, 15.4%) and NX-DCP (*n* = 6 46.2%) were relatively small; therefore, the relationship between serum blood samples and the results of immunohistochemistry was difficult to determine. Further studies with a larger sample size are needed to resolve this issue. Compared to our previous study, the predictive value of vascular invasion in DCP and NX-DCP is relatively high because of the small sample size [17]. This study showed that the serum levels of conventional DCP and NX-DCP were comparable, and immunohistostaining with both markers was similar in this small cohort, though NX-DCP was more convenient for clinical use because of the lower false-positive rate. Moreover, the positive rate for immunohistostaining at vascular invasion sites was more specific with NX-DCP. Therefore, we believe that NX-DCP may be a useful option for routine clinical use. Regarding planning the treatment strategy, patients with high DCP levels should be alerted to the high risk of vascular invasion, and they are not suitable candidates for radiofrequency ablation (RFA) [23] and should be treat by anatomic resection [21].

## Conclusions

This is the first report to find DCP expression at the site of vascular invasion of HCC in an immunohistological study. The results of the present study indicate that NX-DCP promotes the detection of the future malignant potential of HCC, such as the presence of vascular invasion. However, the patient cohort in this study was too small to allow recognition of the value of NX-DCP. This result is likely to contribute to future research into vascular invasion in HCC.

## Data Availability

All data were collected and are kept by the corresponding author. Parts of the data are available by contacting the corresponding author.

## Abbreviations

HCC: Hepatocellular carcinoma
DCP: Des-r-carboxy prothrombin
AFP: Alpha-fetoprotein
NX-DCP: Next-generation DCP
